# Frequent somatic mutations in epigenetic regulators in newly diagnosed chronic myeloid leukemia

**DOI:** 10.1038/bcj.2017.36

**Published:** 2017-04-28

**Authors:** E Togasaki, J Takeda, K Yoshida, Y Shiozawa, M Takeuchi, M Oshima, A Saraya, A Iwama, K Yokote, E Sakaida, C Hirase, A Takeshita, K Imai, H Okumura, Y Morishita, N Usui, N Takahashi, S Fujisawa, Y Shiraishi, K Chiba, H Tanaka, H Kiyoi, K Ohnishi, S Ohtake, N Asou, Y Kobayashi, Y Miyazaki, S Miyano, S Ogawa, I Matsumura, C Nakaseko, T Naoe

**Affiliations:** 1 Department of Hematology, Chiba University Hospital, Chiba, Japan; 2 Department of Pathology and Tumor Biology, Graduate School of Medicine, Kyoto University, Kyoto, Japan; 3 Department of Cellular and Molecular Medicine, Graduate School of Medicine, Chiba University, Chiba, Japan; 4 Department of Clinical Cell Biology and Medicine, Graduate School of Medicine, Chiba University, Chiba, Japan; 5 Department of Hematology and Rheumatology, Faculty of Medicine, Kinki University, Osaka, Japan; 6 Department of Internal Medicine, Hamamatsu University School of Medicine, Hamamatsu, Japan; 7 Department of Hematology, Sapporo Hokuyu Hospital, Sapporo, Japan; 8 Department of Internal Medicine, Toyama Prefectural Central Hospital, Toyama, Japan; 9 Department of Hematology and Oncology, JA Aichi Konan Kosei Hospital, Konan, Japan; 10 Division of Clinical Oncology and Hematology, Department of Internal Medicine, The Jikei University Daisan Hospital, Tokyo, Japan; 11 Department of Hematology, Nephrology and Rheumatology, Akita University Graduate School of Medicine, Akita, Japan; 12 Department of Hematology, Yokohama City University Medical Center, Yokohama, Japan; 13 Laboratory of DNA Information Analysis, Human Genome Center, The Institute of Medical Science, The University of Tokyo, Tokyo, Japan; 14 Department of Hematology and Oncology, Nagoya University Graduate School of Medicine, Nagoya, Japan; 15 Japanese Red Cross Aichi Blood Center, Seto, Japan; 16 Department of Clinical Laboratory Science, Kanazawa University Graduate School of Medical Science, Kanazawa, Japan; 17 Department of Hemato—Oncology, Comprehensive Cancer Center, International Medical Center, Saitama Medical University, Saitama, Japan; 18 Division of Hematology, National Cancer Center Hospital, Tokyo, Japan; 19 Department of Hematology and Molecular Medicine Unit, Atomic Bomb Disease Institute, Nagasaki University Graduate School of Biomedical Sciences, Nagasaki, Japan; 20 Laboratory of Sequence Analysis, Human Genome Center, The Institute of Medical Science, The University of Tokyo, Tokyo, Japan; 21 National Hospital Organization Nagoya Medical Center, Nagoya, Japan

## Abstract

Although tyrosine kinase inhibitors (TKIs) have significantly improved the prognosis of chronic myeloid leukemia (CML), the ability of TKIs to eradicate CML remains uncertain and patients must continue TKI therapy for indefinite periods. In this study, we performed whole-exome sequencing to identify somatic mutations in 24 patients with newly diagnosed chronic phase CML who were registered in the JALSG CML212 study. We identified 191 somatic mutations other than the *BCR-ABL1* fusion gene (median 8, range 1–17). Age, hemoglobin concentration and white blood cell counts were correlated with the number of mutations. Patients with mutations ⩾6 showed higher rate of achieving major molecular response than those<6 (*P*=0.0381). Mutations in epigenetic regulator, *ASXL1*, *TET2*, *TET3*, *KDM1A* and *MSH6* were found in 25% of patients. *TET2* or *TET3*, *AKT1* and *RUNX1* were mutated in one patient each. *ASXL1* was mutated within exon 12 in three cases. Mutated genes were significantly enriched with cell signaling and cell division pathways. Furthermore, DNA copy number analysis showed that 2 of 24 patients had uniparental disomy of chromosome 1p or 3q, which disappeared major molecular response was achieved. These mutations may play significant roles in CML pathogenesis in addition to the strong driver mutation *BCR-ABL1*.

## Introduction

Chronic myeloid leukemia (CML) is a clonal hematopoietic stem cell disorder characterized by a reciprocal translocation between the long arms of chromosomes 9 and 22, resulting in the production of the *BCR-ABL1* fusion gene.^1^ Imatinib, a first-generation tyrosine kinase inhibitor (TKI), has significantly improved the prognosis of CML.^2^ Two second-generation TKIs, nilotinib and dasatinib have been recently approved as frontline treatments for newly diagnosed CML.^3, 4^ These two drugs are more effective than imatinib, and most patients achieve a faster and deeper molecular response than with imatinib.^5, 6^ However, the ability of TKIs to eradicate the CML clone remains uncertain; thus, CML patients may have to continue TKI therapy for indefinite periods. Therefore, a therapeutic goal is the discontinuation of TKIs and development of a curative treatment for CML.

The pathological status of myeloproliferative neoplasms (MPNs) is similar to that of CML because MPNs are also characterized by a very strong driver mutation of *JAK2* V617F. Klampfl *et al.* examined somatic mutations of MPNs, essential thrombocythemia (ET), polycythemia vera (PV) and primary myelofibrosis (PMF) by whole-exome sequencing (WES) and identified mutations to *MPL* and *CALR* in addition to *JAK2* V617F in ET and PMF.^7^ Another group reported the presence of somatic mutations in MPNs, with the *JAK2* mutation being the most frequent, followed by the *CALR* mutation.^8^ In addition to *JAK2* and *CALR*, somatic mutations were also identified in *TET2*, *DNMT3A*, *ASXL1*, and *EZH2* in MPN patients. These genes are reported to be frequently mutated in acute myeloid leukemia (AML) and myelodysplastic syndrome (MDS). Therefore, these mutations may have significant influences on the pathogenesis of MPNs.

The *BCR-ABL1* fusion gene is a strong driver mutation in CML pathogenesis. However, there exists relatively few reports of somatic mutational analysis in CML. Therefore, the objective of the present study was to identify somatic mutations in patients with newly diagnosed CML in the chronic phase (CML-CP) by WES.

## Materials and methods

### Patients

The Japan Adult Leukemia Study Group (JALSG) CML212 study is a multicenter prospective randomized study to compare the cumulative achievement of CMR for adult de novo CML-CP (UMIN Clinical Trials Registry UMIN000007909, http://www.umin.ac.jp/ctrj/). Patients are randomized to either dasatinib or nilotinib. The primary endpoint of the study is a cumulative achievement of CMR by 18 months. Samples from the initial 24 patients enrolled in the JALSG CML212 study between May 2013 and Jan 2014 were analyzed in the present study. We obtained informed consent from all patients to use their samples for banking and molecular analysis, and approval was obtained from the ethics committees of the participating institutes, including the ethical committee of the Graduate School of Medicine, Chiba University (Approval No. 942).

### Wes, deep sequencing and Sanger sequencing

WES and deep sequencing were performed as previously reported.^9, 10, 11^ Briefly, genomic DNA was extracted from peripheral blood mononuclear cells (PBMCs) at the time of CML diagnosis. As a germline control, DNA was obtained from buccal mucosal cells. PBMC DNA was also extracted when a patient achieved a major molecular response (MMR). Whole-exome capture was accomplished by liquid phase hybridization of sonicated genomic DNA with a mean length of 150–200 bp for the bait cRNA library, which was synthesized on magnetic beads (SureSelect Human ALL Exon V5; Agilent Technology, Santa Clara, CA, USA), according to the manufacturer's protocol. The captured targets were subjected to massive sequencing using HiSeq 2000 sequencing system (Illumina, Inc., San Diego, CA, USA) with the pair end 100 bp read option, according to the manufacturer's instructions.

Copy number analysis was performed using in—house pipeline (Shiozawa *et al.* in preparation), in which total copy number of bait regions and common SNPs and allele frequency of heterozygous single-nucleotide polymorphiisms (SNPs) in tumor samples, were used as the input data. The mean coverage of >95% of the target sequences was analyzed at an average depth of more than × 20 (Supplementary Figure S1).

Sanger sequencing against selected variants was performed to validate the mutations identified by WES. We designed the PCR primers to produce PCR products of approximately 1000 bp in length that contained the mutated regions. PCR products were sequenced using the Big Dye Terminator v1.1 cycle sequencing kit (Applied Biosystems, Foster City, CA, USA) and an ABI 3100 Genetic analyzer (Applied Biosystems). The data were analyzed using FinchTV software (Geospiza, Inc., Seattle, WA, USA).

Deep sequencing was also performed for some mutations detected by WES to determine their accurate allele frequencies. Regions containing candidate mutations were amplified from 10–100 ng of DNA samples and prepared for the generation of sequencing libraries using the SureSelectXT2 Reagent kit (Agilent Technologies) according to the manufacturer's instructions. The prepared library was subjected to deep sequencing using a Miseq sequencer (Illumina, Inc.). Obtained Fastq files from the MiSeq were analyzed for image analysis, base calling, and mapping using Strand NGS software (Strand Genomics, Inc., San Francisco, CA, USA). With Strand NGS, the sequencing quality score (−10 × log10 (*P*-value)) calculated with the Bayesian SNP calling algorithm of each base read was used for statistical analyses. For example, a quality score of 20 means an error probability of 1 in 100, and a score of 30 means an error probability of 1 in 1000. We considered bases with quality scores higher than 20 as statistically significant.

### Statistical analyses

Single-nucleotide variants (SNVs) were extracted from whole-exome sequences as somatic mutations. All mutations were compared with published SNP data (dbSNP131, 1000 genome project and an in-house database). Known synonymous SNPs, or SNVs with *P*-values ⩾0.001 compared with the valiant allele frequency (VAF) of peripheral blood leukocytes and oral mucosa by the Fisher's exact test were excluded from further analysis. We also used Fisher's exact test to confirm statistical significance against the results of deep sequencing. Correlations between the number of mutations and clinical factors were identified by the Pearson product—moment correlation coefficient. Receiver operating curve (ROC) analysis was used to establish the number mutations as predicting factor of achieving MMR. Statistical significance between the number of mutations and achieving MMR was identified by Fisher's exact test. These analyses were performed using EZR software (Saitama Medical Center, Jichi Medical University, Saitama, Japan), which is a graphical user interface for R (The R Foundation for Statistical Computing, Vienna, Austria).^12^ Gene ontology (GO) analysis was used to evaluate functional enrichment in GO terms among mutated genes detected by WES. Sequencing reads were aligned to the human genome reference (hg19) using the Shirokane 2 supercomputer system, and Fisher's exact test was used to calculate the *P-*values of GO analysis.

## Results

### Patient characteristics

The patient characteristics are shown in Table 1. The median age of the patients (18 males and 6 females) at diagnosis was 54.5 years (range, 23–77 years). The mean white blood cell count was 96.2±127.7 × 10^9^ cells per liter, and the mean hemoglobin concentration was 12.7±2.6 g dl^−1^. Two patients (8.3%) had additional mutations besides t(9;22) that were detected by the G-band staining method. The median international scale (IS) for %*BCR-ABL1/ABL1* was 56.9±28.4%, excluding two patients with a minor *BCR-ABL1* mutation. Two patients (No. 4 and 7) dropped out this trial because of side effects and patient's reasons. Of the remaining 22 patients, 18 patients (82%) achieved MMR at 2 years, while four patients did not (Figure 2).

### Summary of mutated genes

The WES results identified 191 somatic gene mutations in 24 patients (Supplementary Table S1). The number of mutations for each patient ranged from 1 to 17, with a median of 8. Correlation coefficients were calculated between the number of mutations and clinical factors, which revealed mild positive correlations with age (*r*=0.50, *P*<0.05) and hemoglobin concentration (*r*=0.48, *P*<0.05), with a moderate negative correlation with white blood cell count (*r*=−0.53, *P*<0.067). There were no correlations between the level of major *BCR-ABL/ABL*% IS (*r*=0.12), EUTOS score (*r*=−0.33), or Sokal score (*r*=−0.01) with the number of mutations. ROC analysis established 6 mutations as a cutoff level for comparing the rate of achieving MMR with an area under the curve of 0.69. Patients with mutations ⩾6 showed higher rate of achieving MMR (*n*=15 put of 17, 88.2%) than those<6 (3 out of 7, 42.9% *P*=0.0381).

**Figure 1:**
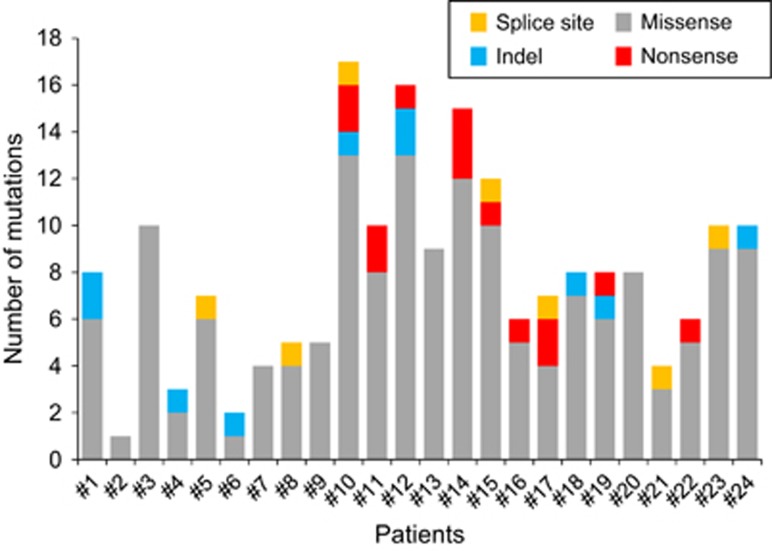
Somatic mutations in CML-CP detected by WES. Number of validated somatic changes and the types of mutation in 24 patients are shown in different colors. Splice site mutations are shown in yellow, nonsense in red, indel in blue and missense in gray.

Among the 191 mutations, 166 were missense, which included four splice-site mutations. The remaining mutations were either frameshift (*n*=8) or non-frameshift (*n*=3) indels or nonsense (*n*=14). The types of somatic changes identified in each patient are shown in Figure 1.

**Figure 2:**
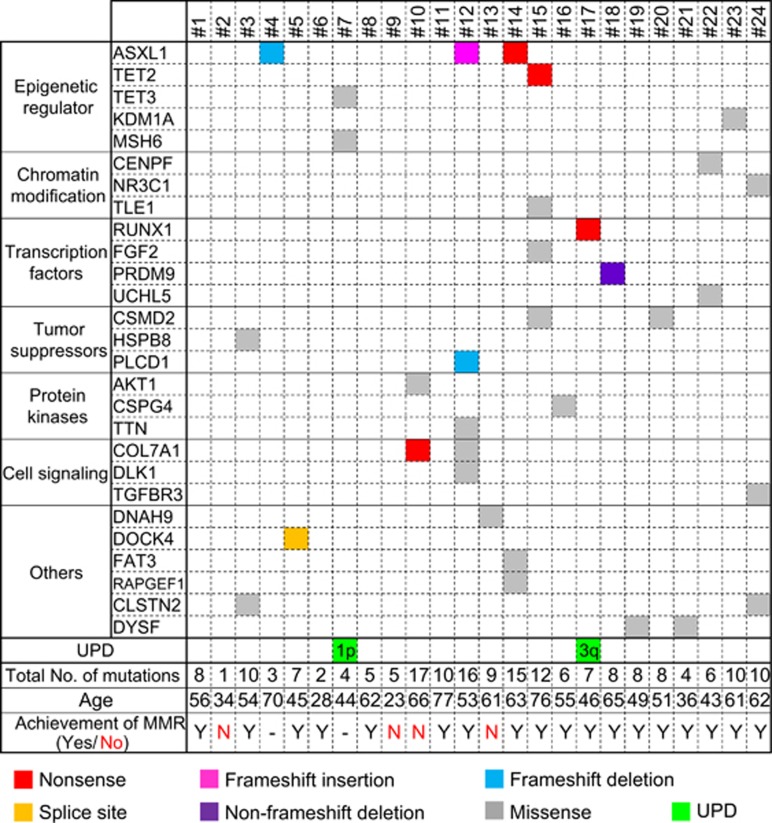
Summary of somatic mutations of interest and DNA copy number alterations detected by WES in 24 CML cases. Of all 191 somatic mutations identified by WES, recurrent mutations or mutations previously reported as being related to hematological malignancies were identified. Nonsense mutations are shown in red, missense in gray, frameshift insertions or deletions, and non-frameshift deletions in pink, blue, and purple, respectively. DNA copy number alterations were detected by WES, and UPDs are shown in green. The number on the cells describes the chromosome and its arm with UPD. Patients' age at diagnosis and achievement of MMR are described in the last.

**Figure 3:**
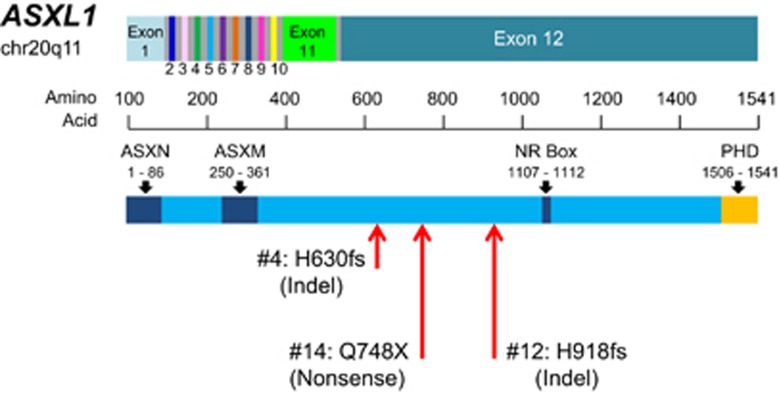
Mutations found in ASXL1 gene. *ASXL1* exist on chromosome 20q11. Three mutations in *ASXL1* were expressed in exon 12 showing with red arrows; all of them were loss-of-function mutations. The C-terminal of exon 12 contains a PHD finger that is a structural motif found in nuclear proteins and has reported to be involved in transcriptional regulation, chromatin modifications, and histone demethylation.

We found mutations in epigenetic regulator, *ASXL1*, *TET2*, *TET3*, *KDM1A* and *MSH6* in 6 out of 24 patients (25%, Figure 2). *ASXL1* was mutated in three cases, all of them were found within exon 12 (Figure 3). *TET2*, *TET3*, *KDM1A* and *MSH6* were mutated in one case each. There were recurrent mutations expressed in *CLSTN2*, *COL7A1*, *CSMD2* and *DYSF*. Recurrent mutations or mutations previously reported to be related with hematological malignancies such as *RUNX1* or *AKT1* were validated using Sanger sequencing or deep sequencing and summarized into 7 functional groups with DNA copy number alterations in Figure 2 and Table 2.

### Copy number alterations by WES

**Figure 4:**
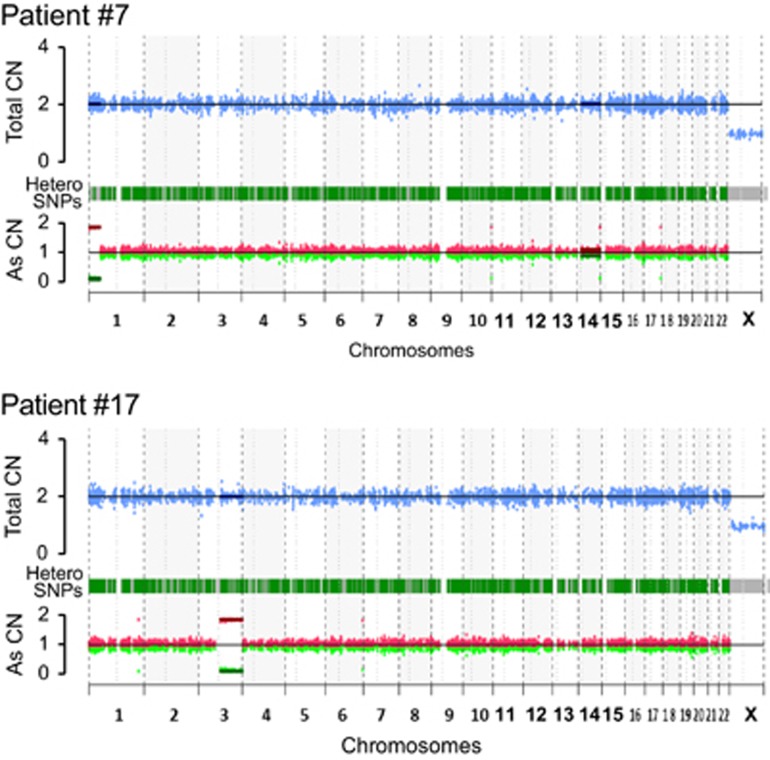
DNA copy number alteration in two cases with UPD, detected by WES. Patients No. 7 and 17 had UPD in chromosomes 1p and 3q, respectively. Copy number alterations were analyzed by comparing the total copy number with the allele—specific copy number. Blue dots on the upper line indicate the total copy number, while red and green dots on the lower line represent each the allele-specific copy number of hetero SNPs.

DNA copy number alterations were analyzed by comparing the total copy number with the allele specific copy number detected by WES. Two patients (No. 7 and 17) were found to have uniparental disomy (UPD) of chromosomes 1p and 3q, respectively (Figure 4). *SFPQ* on 1p of patient #7 was mutated, while there was no mutation of 3q in patient No. 17. The VAF of mutated *SFPQ* was 0.47.

Heterozygous SNPs in *ABCC5*, *C3orf37*, *IQCJ* and *PRR23A* of chromosome 3q were discovered in buccal cells from patient No. 17; therefore, we performed Sanger sequencing with a DNA samples derived from PBMCs collected at diagnosis and after achieving MMR. The heterozygous SNPs of these four genes were all homozygously mutated at diagnosis and then returned to heterozygous mutations once MMR was achieved. These results suggest that UPD of chromosome 3q in patient No. 17 disappeared with TKI treatment.

### Results of GO analysis

**Figure 5:**
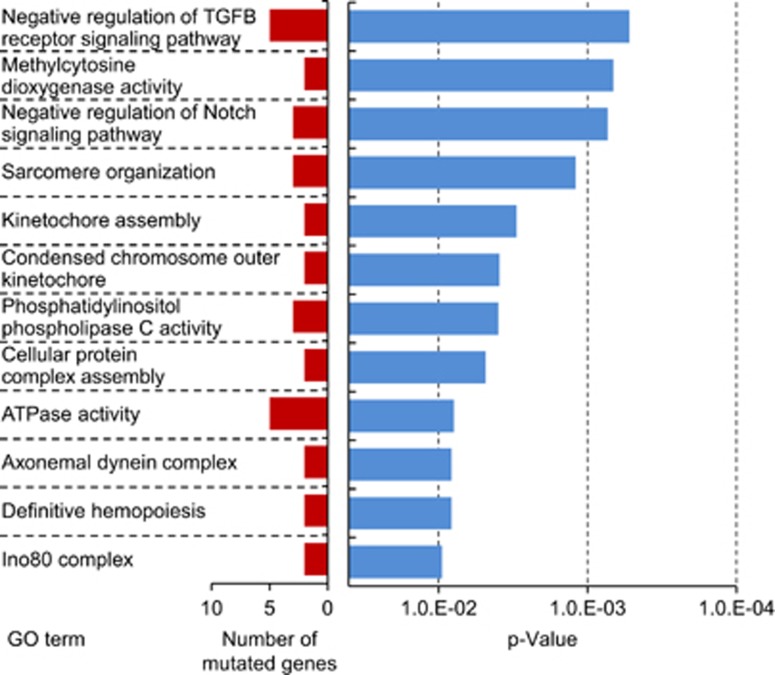
Results of GO analysis. GO analysis was performed to evaluate functional enrichment in GO terms among mutated genes detected by WES. Sequencing reads were aligned to the human genome reference (hg19). GO terms with lower *P* values calculated using the Fisher's exact test and higher frequency of annotation are shown.

GO analysis, performed to evaluate functional enrichment in GO terms among mutated genes detected by WES, found that mutated genes were mostly enriched with cell signaling and cell division pathways. Among the results, GO terms with the *P*-value <0.01 and recurrently annotated were listed in Supplementary Table S2. Moreover, some GO terms thought to be related with tumorigenesis were selected on Figure 5.

### Deep sequencing with MMR samples

Paired samples obtained from PBMCs both at diagnosis and after achieving MMR were available for three patients with mutated *ASXL1* (patient No. 12), *RUNX1* (patient No. 17), and *KDM1A* (patient No. 23) at diagnosis. We performed deep sequencing with these paired DNA samples to detect consecutive changes of mutations and found transition of VAF in these three mutations. These mutations of *ASXL1*, *RUNX1* and *KDM1A* all disappeared once MMR was achieved (Supplementary Figure S2).

## Discussion

We identified 191 somatic mutations, other than the *BCR-ABL1* fusion gene, by WES in 24 newly diagnosed CML-CP patients. GO analysis revealed that the mutated genes were significantly enriched with cell signaling and cell division pathways. This result suggests that the cell signaling or cell division pathway was activated at CML onset. Some mutations in epigenetic regulator, *ASXL1*, *TET2*, *TET3*, *KDM1A* and *MSH6* were found in 25% of patients. Moreover, *AKT1*, a kinase activator and *RUNX1*, a promotor of transcriptional regulation, have been reported as frequently mutated genes in AML/MDS and MPNs, and were mutated in one patient each.

DNA methylation has reported to be associated with pathogenesis of CML.^13^ Amabile *et al.* reported that aberrant DNA methylation of CML in murine models.^14^ DNA methylation changes were driven by *BCR-ABL1* expression and contributed to the disease evolution. DNA methylation changes can act as a secondary event and contribute to leukemia formation, and using 5-Azacytidine, a DNA methyltransferase inhibitor, prolonged survival rate of murine model of CML. Moreover, methylation of additional genes other than *BCR-ABL1* has reported in TKI-resistant CML patients, and is associated with their prognosis.^15^ Taken together, epigenetic regulation may play important roles for the pathology of CML.

Three loss-of-function mutations (frameshift insertion, deletion or nonsense mutation) found in *ASXL1* all existed within exon 12. *ASXL1* has well-known roles in histone modification and as a putative tumor suppressor gene that is often reported to be mutated in hematological malignancies. In MDS/MPN patients, *ASXL1* mutations were concentrated within exon 12.^16, 17^ Moreover, Boultwood *et al.* performed sequencing analysis of *ASXL1* within exon 12 of 41 pre-imatinib CML patients and identified *ASXL1* mutations in six cases.^18^ Frameshift or nonsense mutations in exon 12 of *ASXL1* should lead to the truncation of the protein and removal the C-terminal, which contains a PHD finger that is a structural motif found in nuclear proteins and has reported to be involved in transcriptional regulation, chromatin modifications and histone demethylation (Figure 5).^17, 19, 20, 21, 22^ A PHD finger recognizes the methylation status of histone lysine residues, such as histone H3 lysine 4, and its mutation has been reported in many diseases, including hematological malignancies.^23^ Taken together, the loss-of-function mutation of *ASXL1* leads to PHD finger dysregulation, which may be related to tumorigenesis in CML. Moreover, deep sequencing revealed that the *ASXL1* mutation of patient #12 at diagnosis had disappeared once MMR was achieved, while Boultwood *et al.* also reported *ASXL1* mutations in four patients with CML-CP.^18^ These results suggest that an *ASXL1* mutation indicate the disease state or prognosis of CML.

*TET2* is one of the epigenetic regulator genes and frequently mutated in hematological malignancies, including CML.^24^ Wang *et al.* performed systematic mutation analysis by WES of PV and found recurrent somatic mutations in *ASXL1*, *DNMT3A*, *TET2* and *SF3B1.*^25^ Ortmann *et al.*^26^ reported *TET2* mutations in *JAK2* mutation-positive MPN patients to clarify the effect of mutation order on disease phenotype and progression. They performed clonogenic analysis and detected, which patient is ‘*TET2* first' or ‘*JAK2* first.' The majority of bone marrow progenitor cells were common myeloid progenitors in ‘*TET2* first' patients, while megakaryocyte-erythroid progenitors were predominant in ‘*JAK2* first' patients. These reports suggest that the existence of another somatic mutation in addition to the strong driver mutation *BCR-ABL1* may influence the disease phenotype. *TET3* shares significant sequence homology with *TET1* and *TET2*. Sequencing analysis of *TET3* in myeloid malignancy, excluding CML, revealed no mutations among 96 myeloproliferative neoplasm patients.^27^ However, because *TET2* and *TET3* have overlapping requirements in hematopoietic stem cell emergence, the *TET3* mutation may play a role in CML.^28^

*KDM1A* (also known as *LSD1*) is associated with the maintenance and differentiation of HSCs by demethylation of H3K4me2.^29^ *KDM1A* is upregulated in prostate cancer or neuroblastoma, and its expression has been reported to correlate with adverse outcome or inversely correlate with differentiation in tumors. *KDM1A* has been also reported as an essential regulator of leukemia stem cell potential in a murine model of human *MLL-AF9* leukemia,^30^ with persistence of expression in associated oncogenic signaling, thereby preventing differentiation and apoptosis of leukemic cells. Moreover, treatment with the novel *KDM1A* antagonist significantly improved the survival of murine model of human AML, with inducing differentiation and apoptosis of leukemic cells.^31^ These results suggest that *KDM1A* may be related with maintenance of leukemic cell as an epigenetic regulator.

*MSH6* is an essential component of the DNA mismatch repair mechanism and has been proposed to interact as an epigenetic regulator.^32^ Loss-of-function mutation was reported in relapsed ALL patients^33^ and the mutation leads to constitutional mismatch repair deficiency syndrome, which is characterized by the development of childhood cancers, mainly hematological malignancies.^34^ Taken together, epigenetic regulation may play important roles against the etiology of CML.

Recurrent somatic mutations in *COL7A1*, *CSMD2*, *CLSTN2* and *DYSF* were also found in two patients each. It has been reported that *COL7A1* expression was significantly upregulated in cancer stem cells in solid tumors by the positive stimulation of *TGFB1* signaling.^35^ *TGFB* is a critical regulator of Akt activation in leukemia-initiating cells and controls FOXO3A localization in CML, which is responsible for maintaining leukemia-initiating cells.^36^ A *CSMD2* mutation has not yet been reported in hematological malignancies, but it is a candidate tumor suppressor gene in pancreatic and colorectal cancers. Hypermethylation of *CSMD2* in pancreatic cancer^37^ or its low expression in colorectal cancer was significantly associated with differentiation, lymphatic invasion, tumor size and overall survival.^38^ *CLSTN2* encodes the synaptic protein calsyntenin 2 and is related to human memory and hippocampal function.^39^ *DYSF* is highly expressed in the skeletal muscle and has been suggested to be involved in membrane regeneration and repair. Recently, *DYSF* was also reported to be expressed in monocytes and its depletion impaired cell adhesion.^40^ However, mutations in these two genes *DYSF* are rarely reported in cancer.

Furthermore, there are several genes that have been previously reported in association with hematological malignancies. *RUNX1* is a transcription factor that controls myeloid differentiation. Many reports have revealed that *RUNX1* is mutated in the blastic crisis (BC) stage of CML (CML-BC), indicating that its mutation may affect CML progression.^41, 42^ *RUNX1*-deficient mice developed a mild myeloproliferative phenotype characterized by an increase in peripheral blood neutrophils, myeloid progenitor populations and extramedullary hematopoiesis.^43^ Furthermore, Zhao *et al.*^44^ reported transduction of both H78Q and V91fs—ter94 variants of *RUNX1* into 32D cells or BCR-ABL-harboring murine cells, which resulted in disrupted myeloid differentiation and induction of a BC or accelerated phase-like phenotype in mice. These results suggest that *RUNX1* alterations contribute to CML onset and progression.

In this study, copy number analysis by WES revealed UPD in chromosome 1p or 3q of two patients. Boultwood *et al.*^18^ also performed SNP array analysis of samples from 41 pre-imatinib CML-CP or -BC patients. A total of 65 regions of UPD were detected in 29 of 41 patients.

Eight recurrent regions of UPD were observed, and paired analysis of CP and BC samples identified two regions of UPD only in two patients in the BC phase. In our study, UPD on chromosome 3q in patient #17 disappeared with TKI treatment. Taken together, these data suggest that UPD is associated with disease evolution in CML. UPD could result from the mitotic recombination between chromatids of homologous chromosomes, which sometimes leads to transition from heterozygosity to homozygosity of each mutation. Mutations to *JAK2* in PV,^45^ *CBL* in MPN,^46^ *CEBPA* in AML^47^ and *RUNX1* in MDS/AML^48^ were found in association with UPD regions.

In this study, there were no significant mutations with UPD lesions on chromosome 1p and 3q, but there exist *NRAS* or *JAK1* on chromosome 1p, while *BCL6*, *GATA2* or *TP63* on chromosome 3q. Some kinds of congenital disorders, such as Prader–Willi syndrome, are reported to have UPDs in certain imprinting regions.^49^ Dysfunction of imprinting genes caused by UPDs results in a disease onset. UPDs may affect the expression of genes.

In summary, we performed WES using samples collected from 24 newly diagnosed CML-CP patients. Although many recent studies have reported somatic mutations by next-generation sequencing in AML, MDS and MPN, this is the first report of somatic mutations in multiple cases of CML-CP. We found mutations of epigenetic regulator, *ASXL1*, *TET2*, *TET3*, *KDM1A* and *MSH6* in 25% of patients, and also *AKT1* and *RUNX1* in each patient. Besides these mutations, multiple novel recurrent mutations previously reported in association with hematological malignancies were also found. Further analyses of long-term follow-up, functional analysis of these candidate genes or detecting transition of these mutations by deep sequencing may predict whether somatic mutations other than BCR-ABL1 can be related to their prognosis such as therapeutic resistance or relapse.

## Figures and Tables

**Table 1:** Patient characteristics

*Median age (year)*	54.5	(Range, 23–77)
*Sex*		
Male	18	75%
Female	6	25%
		
*EUTOS score*^a^	36.2	(Range, 3.5–119.0)
Low	20	83.3%
High	4	16.7%
		
*Sokal score*^b^	0.84	(Range, 0.58–11.42)
Low	11	45.8%
Int	9	37.5%
High	4	16.7%
Time from diagnosis to treatment (days)	12.5	(Range, 4–37)
IS *BCR-ABL1/ABL1* (%)^c^	56.9±28.4	
WBCs (per μl)	96200±127700	
Hb (g dl^−1^)	12.7±2.6	
Platelet (× 10^a^9/l)	533±546	
		
*Additional chromosomal mutations*		
Yes	2 Patient #2: Patient #4:	8.3% t(8;17)(q11.2;q23) −Y
No	22	91.7%
		
*Achieving MMR at 2 years*		
Yes	18	75.0%
No^d^	6	25.0%

Abbreviations: MMR, major molecular response; WBCs, white blood cells.

a EUTOS score:⩽87: low risk, >87: high risk.

b Sokal score:⩽0.8: low risk, 0.8–1.2: intermediate risk, >1.2: high risk.

c IS %*BCR-ABL/ABL1* (%): % of *BCR-ABL1* mRNA International Scale. Two patients with minor *BCR-ABL1* mutations were excluded.

d 4 patients were still not achieving MMR and 2 patients were dropped out from this study.

**Table 2:** Representative mutated genes

*UPN*	*Age (years)*	*Gene*	*Function*	*Amino acid change*	*Chr*	*Ref*	*Obs*	*VAF PB*
#4	70	ASXL1	Indel	NM_015338:p.H630f s	chr20	CACCACTGCCATAGAGAGGCGGC	—	0.17
#12	53	ASXL1	Indel	NM_015338:p.H918fs	chr20	-	A	0.29
#14	63	ASXL1	Nonsense	NM_015338:p.Q748X	chr20	C	T	0.50
#10	66	AKT1	Missense	NM_001014431:p.I180F	chr14	T	A	0.51
#15	76	TET2	Nonsense	NM_001127208:p.R544X	chr4	C	T	0.45
#7	44	TET3	Missense	NM_144993:p.A128T	chr2	G	A	0.51
#17	46	RUNX1	Nonsense	NM_001001890:p.S114X	chr21	G	T	0.15
#18	65	PRDM9	Indel	NM_020227:p.11_12del	chr5	AGA	—	0.33
#10	66	COL7A1	Nonsense	NM_000094:p.K1859X	chr3	T	A	0.50
#12	53	COL7A1	Missense	NM_000094:p.E1167K	chr3	C	T	0.40
#15	76	CSMD2	Missense	NM_052896:p.V808I	chr1	C	T	0.51
#20	51	CSMD2	Missense	NM_052896:p.T27M	chr1	G	A	0.38

Abbreviations: Chr, chromosome; PB, peripheral blood cells; Ref, reference; Obs, observation; VAF, variant allele frequency.
