# Supplementary material for: Frequent somatic mutations in epigenetic regulators in newly diagnosed chronic myeloid leukemia
Source: Blood Cancer J. 2017 Apr 28;7(4):e559–. doi: 10.1038/bcj.2017.36 (PMC5436079; doi:10.1038/bcj.2017.36)
Supplement: Supplementary Materials [file bcj201736x1.docx]

**Supplementary materials**

**Supplementary figures**

Supplementary figure S1. Mean coverage of WES in 24 paired tumor/control samples

Supplementary figure S2. Deep sequencing with MMR samples

**Supplementary tables**

Supplementary table S1. Results of 191 somatic mutations detected by WES

Supplementary table S2. Results of GO analysis with *p* value < 0.01

**Supplementary Figure S1. Mean coverage of WES in 24 paired tumor/control samples**

Tumor samples were obtained from PBMCs at the time of diagnosis of CML and control samples as germline cells were obtained from buccal mucosa. Blue bar shows a depth of more than ×30, red of more than ×20, and green of more than ×10. The mean coverage of more than 95% of the target sequences was analyzed at an average depth of more than ×20.

#n-B; sample of buccal cells from patient #n

#n-T; sample of peripheral blood mononuclear cells at diagnosis (defined as ‘tumor cells’) from patient #n

**Supplementary Figure S2. Deep sequencing with MMR samples**

Paired samples obtained from PBMCs both at diagnosis and after achieving MMR were available for three patients with mutated *ASXL1* (patient #12), *RUNX1* (patient #17), and *KDM1A* (patient #23) at diagnosis.


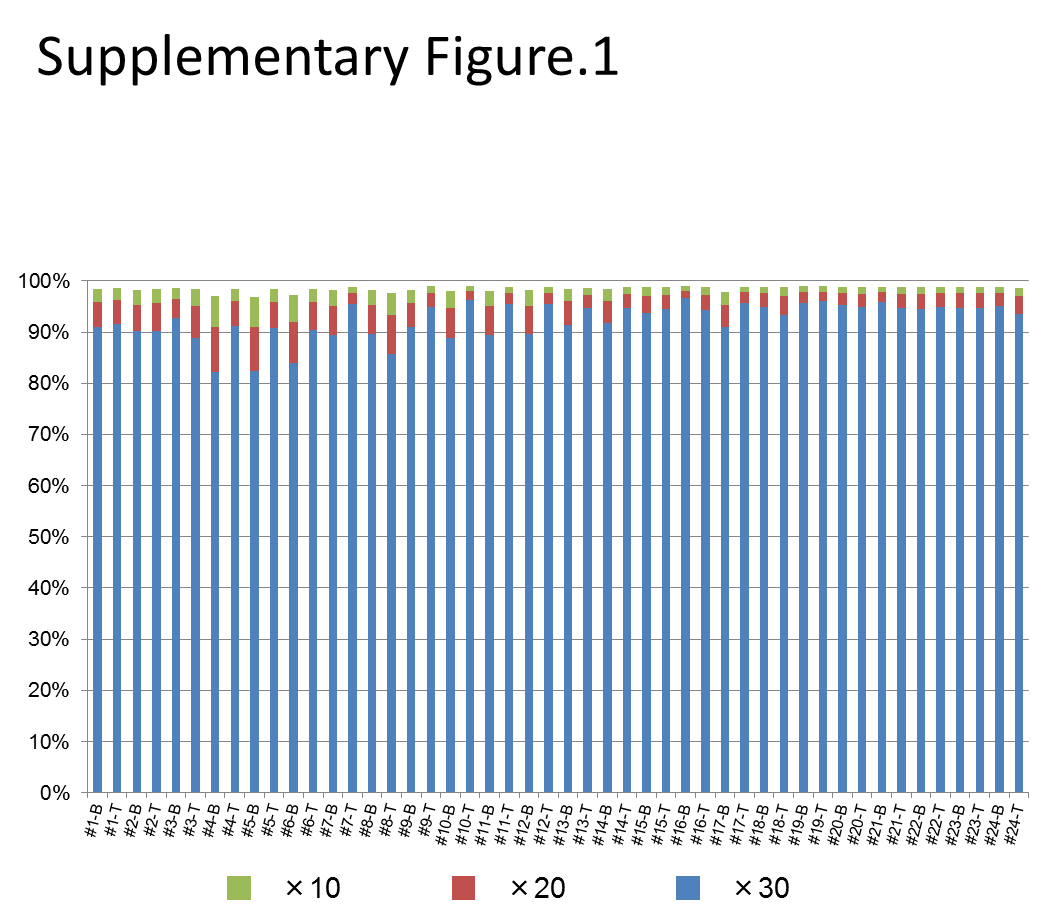


**
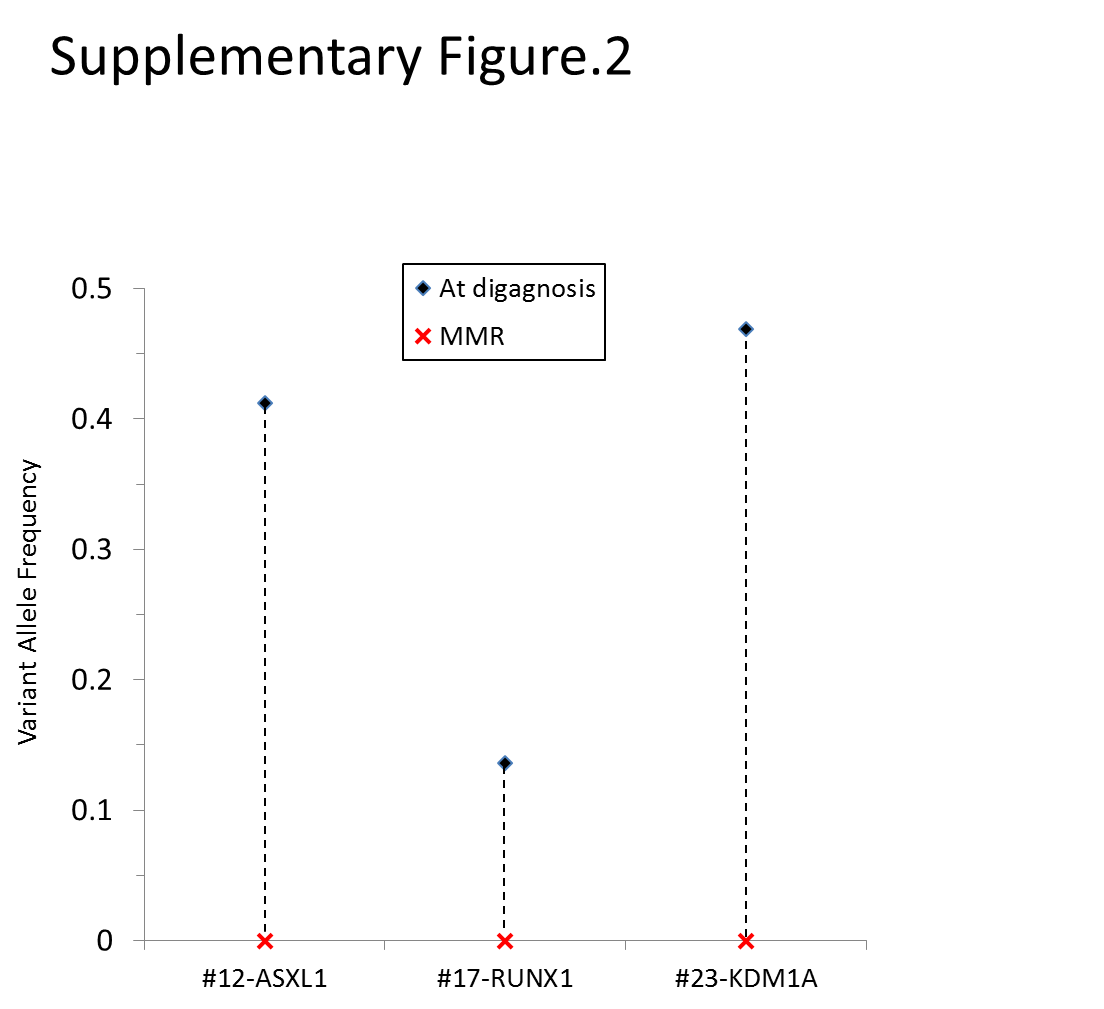
**

**Supplementary Table S1. Results of 191 somatic mutations detected by WES**

| UPD | Annotated  Gene | Mutation  Type | RefSeq | Allele Change | Amino Acid  Change | Chr | Position |
| --- | --- | --- | --- | --- | --- | --- | --- |
| #1 | *ARHGEF33* | Indel | NM_001145451 | */-T | S820fs | chr2 | 39193261 |
| #1 | *ARHGEF33* | Missense | NM_001145451 | T>C | S820P | chr2 | 39193261 |
| #1 | *GPR98* | Missense | NM_032119 | G>A | R1553H | chr5 | 89954001 |
| #1 | *HIST1H4E* | Indel | NM_003545 | */+T | G12fs | chr6 | 26204907 |
| #1 | *MYH6* | Missense | NM_002471 | C>T | A111T | chr14 | 23874850 |
| #1 | *NDUFA13* | Missense | NM_015965 | T>A | V101E | chr19 | 19638566 |
| #1 | *SRCAP* | Missense | NM_006662 | C>T | P1671S | chr16 | 30735756 |
| #1 | *TG* | Missense | NM_003235 | A>G | T462A | chr8 | 133899001 |
| #2 | *HTRA1* | Missense | NM_002775 | A>G | T299A | chr10 | 124266324 |
| #3 | *CLSTN2* | Missense | NM_022131 | G>A | G776R | chr3 | 140281766 |
| #3 | *ENPEP* | Missense | NM_001977 | G>A | A666T | chr4 | 111464222 |
| #3 | *HERPUD1* | Missense | NM_001010990 | G>T | V171F | chr16 | 56973838 |
| #3 | *HEY2* | Missense | NM_012259 | C>T | T228M | chr6 | 126080617 |
| #3 | *HSPB8* | Missense | NM_014365 | C>A | P90H | chr12 | 119617386 |
| #3 | *MAPKBP1* | Missense | NM_014994 | A>G | H923R | chr15 | 42113831 |
| #3 | *MYO1B* | Missense | NM_001130158 | G>A | R771Q | chr2 | 192265124 |
| #3 | *ODF1* | Missense | NM_024410 | C>T | P208L | chr8 | 103572982 |
| #3 | *SLC12A4* | Missense | NM_001145962 | C>T | G648S | chr16 | 67981603 |
| #3 | *VWCE* | Missense | NM_152718 | C>G | V163L | chr11 | 61053840 |
| #4 | *ASXL1* | Indel | NM_015338 | */-CACCACTGCCATAGAGAGGCGGC | H630fs | chr20 | 31022403 |
| #4 | *EVI2A* | Missense | NM_014210 | T>C | N130S | chr17 | 29645643 |
| #4 | *TENC1* | Missense | NM_015319 | C>A | P233T | chr12 | 53449445 |
| #5 | *C6orf132* | Missense | NM_001164446 | G>A | A794V | chr6 | 42073269 |
| #5 | *DOCK4* | Missense/  splice site | NM_014705 | C>T | R1296Q | chr7 | 111407089 |
| #5 | *EHD1* | Missense | NM_006795 | G>C | L481V | chr11 | 64621969 |
| #5 | *ESRP1* | Missense | NM_001034915 | C>T | A386V | chr8 | 95680402 |
| #5 | *FRG2/LOC100288255* | Missense | NM_001005217 | C>A | S174I | chr4 | 190947032 |
| #5 | *KCNU1* | Missense | NM_001031836 | A>G | I114V | chr8 | 36661569 |
| #5 | *TPPP* | Missense | NM_007030 | C>T | V96M | chr5 | 677890 |
| #6 | *APOL1* | Indel | NM_001136541 | */-GTG | 215_216del | chr22 | 36661581 |
| #6 | *TDRD6* | Missense | NM_001010870 | C>T | P1080S | chr6 | 46659103 |
| #7 | *MSH6* | Missense | NM_000179 | T>C | V762A | chr2 | 48027407 |
| #7 | *OR5AP2* | Missense | NM_001002925 | C>T | D25N | chr11 | 56409843 |
| #7 | *SFPQ* | Missense | NM_005066 | G>A | P249L | chr1 | 35657905 |
| #7 | *TET3* | Missense | NM_144993 | G>A | A128T | chr2 | 74273831 |
| #8 | *ACTL6A* | Missense | NM_004301 | T>G | I381M | chr3 | 179304354 |
| #8 | *BSCL2* | Missense | NM_001122955 | C>T | R456H | chr11 | 62457861 |
| #8 | *CACNA1B* | Missense  /splice site | NM_000718 | C>A | T323K | chr9 | 140846727 |
| #8 | *CLDND2* | Missense | NM_152353 | G>A | S129F | chr19 | 51870755 |
| #8 | *DLK2* | Missense | NM_023932 | G>A | P252L | chr6 | 43418674 |
| #9 | *CD27* | Missense | NM_001242 | C>G | P260R | chr12 | 6560554 |
| #9 | *CPB2* | Missense | NM_016413 | G>T | T324N | chr13 | 46629902 |
| #9 | *IL17RD* | Missense | NM_017563 | G>T | R573S | chr3 | 57132014 |
| #9 | *KBTBD8* | Missense | NM_032505 | A>C | I309L | chr3 | 67054316 |
| #9 | *SYPL2* | Missense | NM_001040709 | C>T | T206M | chr1 | 110020600 |
| #10 | *AEN* | Indel | NM_022767 | */-C | C284fs | chr15 | 89173399 |
| #10 | *AKT1* | Missense | NM_001014431 | T>A | I180F | chr14 | 105241442 |
| #10 | *CASR* | Missense | NM_000388 | G>A | G363S | chr3 | 121980969 |
| #10 | *COL7A1* | Nonsense | NM_000094 | T>A | K1859X | chr3 | 48614346 |
| #10 | *DGKG* | Missense | NM_001080745 | C>T | A377T | chr3 | 185983059 |
| #10 | *EDC4* | Missense | NM_014329 | A>G | H881R | chr16 | 67915173 |
| #10 | *EIF4G1* | Missense | NM_004953 | A>G | N449S | chr3 | 184040875 |
| #10 | *LRRC56* | Missense | NM_198075 | A>C | K141Q | chr11 | 549996 |
| #10 | *LRRTM4* | Missense | NM_001134745 | C>T | V394I | chr2 | 77745815 |
| #10 | *MUC2* | Missense | NM_002457 | C>T | T1606M | chr11 | 1092998 |
| #10 | *PPP1R15A* | Nonsense | NM_014330 | C>T | R121X | chr19 | 49376851 |
| #10 | *PTCHD3* | Missense | NM_001034842 | G>C | P104A | chr10 | 27702870 |
| #10 | *R3HDM2* | Missense | NM_014925 | C>T | G912E | chr12 | 57648752 |
| #10 | *TEAD3* | Missense | NM_003214 | G>A | S91F | chr6 | 35448147 |
| #10 | *THOC1* | Missense | NM_005131 | C>T | D370N | chr18 | 225118 |
| #10 | *SIPA1L1* | Splice site | NM_015556 | G>T | - | chr14 | 72090954 |
| #10 | *ZNF546* | Missense | NM_178544 | G>A | V193I | chr19 | 40519754 |
| #11 | *DDX27* | Missense | NM_017895 | A>G | N31D | chr20 | 47835983 |
| #11 | *HM13* | Nonsense | NM_178580 | C>T | Q389X | chr20 | 30156011 |
| #11 | *LHFPL3* | Missense | NM_199000 | G>T | K178N | chr7 | 104377210 |
| #11 | *NGF* | Missense | NM_002506 | C>G | R75S | chr1 | 115829192 |
| #11 | *OOEP* | Nonsense | NM_001080507 | G>A | R67X | chr6 | 74079100 |
| #11 | *OR8K1* | Missense | NM_001002907 | A>G | D186G | chr11 | 56114071 |
| #11 | *PHLDB2* | Missense | NM_001134438 | C>T | R339W | chr3 | 111603939 |
| #11 | *PLXNA4* | Missense | NM_020911 | C>T | G1661R | chr7 | 131831343 |
| #11 | *THSD4* | Missense | NM_024817 | C>T | T631M | chr15 | 72030332 |
| #11 | *TTC23L* | Missense | NM_144725 | C>T | A148V | chr5 | 34863066 |
| #12 | *ASXL1* | Indel | NM_015338 | */+A | H918fs | chr20 | 31023269 |
| #12 | *BAG5* | Missense | NM_001015048 | G>A | P174L | chr14 | 104026981 |
| #12 | *BCAN* | Nonsense | NM_198427 | G>A | W669X | chr1 | 156622749 |
| #12 | *COL7A1* | Missense | NM_000094 | C>T | E1167K | chr3 | 48623816 |
| #12 | *DLK1* | Missense | NM_003836 | G>T | A213S | chr14 | 101200718 |
| #12 | *GLT25D1* | Missense | NM_024656 | T>C | V165A | chr19 | 17678219 |
| #12 | *GOLGA4* | Missense | NM_002078 | G>C | A1935P | chr3 | 37369180 |
| #12 | *LAX1* | Missense | NM_001136190 | G>A | M61I | chr1 | 203740526 |
| #12 | *NPNT* | Missense | NM_001033047 | A>T | K412M | chr4 | 106879703 |
| #12 | *OR5B12* | Missense | NM_001004733 | G>A | L64F | chr11 | 58207435 |
| #12 | *PLCD1* | Indel | NM_001130964 | */-C | D396fs | chr3 | 38051636 |
| #12 | *PCLO* | Missense | NM_014510 | C>T | A3287T | chr7 | 82580045 |
| #12 | *RPH3A* | Missense | NM_014954 | A>T | E52D | chr12 | 113285585 |
| #12 | *TIPARP* | Missense | NM_001184717 | A>T | D299V | chr3 | 156396382 |
| #12 | *TMEM56/*  *TMEM56-*  *RWDD3* | Missense | NM_001199679 | C>T | A95V | chr1 | 95615802 |
| #12 | *TTN* | Missense | NM_003319 | C>T | R14410H | chr2 | 179440435 |
| #13 | *C1orf94* | Missense | NM_001134734 | G>C | C160S | chr1 | 34662984 |
| #13 | *CENPC1* | Missense | NM_001812 | A>C | S769R | chr4 | 68358699 |
| #13 | *DNAH9* | Missense | NM_001372 | C>A | A1355E | chr17 | 11593203 |
| #13 | *FAM47A* | Missense | NM_203408 | G>A | R544C | chrX | 34148766 |
| #13 | *MB21D2* | Missense | NM_178496 | A>G | .I415T | chr3 | 192516407 |
| #13 | *MYH1* | Missense | NM_005963 | C>T | R1140H | chr17 | 10404746 |
| #13 | *OR1N2* | Missense | NM_001004457 | C>T | A65V | chr9 | 125315642 |
| #13 | *PGK1* | Missense | NM_000291 | G>A | G167D | chrX | 77372891 |
| #13 | *SPEF2* | Missense | NM_024867 | G>A | R1452H | chr5 | 35779356 |
| #13 | *WDR62* | Missense | NM_001083961 | C>T | T1114I | chr19 | 36593855 |
| #14 | *ABCC9* | Missense | NM_005691 | C>T | R304H | chr12 | 22065906 |
| #14 | *ACAD11* | Missense | NM_032169 | G>A | R685C | chr3 | 132280010 |
| #14 | *ASB18* | Missense | NM_212556 | G>T | L458I | chr2 | 237103544 |
| #14 | *ASXL1* | Nonsense | NM_015338 | C>T | Q748X | chr20 | 31022757 |
| #14 | *ELOVL2* | Missense | NM_017770 | C>T | A223T | chr6 | 10990034 |
| #14 | *FAT3* | Missense | NM_001008781 | T>C | I1132T | chr11 | 92257902 |
| #14 | *KCTD13* | Missense | NM_178863 | C>G | G81A | chr16 | 29937113 |
| #14 | *LMX1A* | Missense | NM_001174069 | G>A | L129F | chr1 | 165218756 |
| #14 | *N6AMT2* | Missense | NM_174928 | G>A | R162W | chr13 | 21306004 |
| #14 | *ORC3* | Missense | NM_012381 | T>A | V134D | chr6 | 88315713 |
| #14 | *PCDHA10* | Missense | NM_018901 | C>T | P244L | chr5 | 140236364 |
| #14 | *PLEKHG3* | Nonsense | NM_015549 | C>T | Q1125X | chr14 | 65210302 |
| #14 | *RAPGEF1* | Missense | NM_005312 | G>A | P613S | chr9 | 134497200 |
| #14 | *STXBP5L* | Nonsense | NM_014980 | C>T | R904X | chr3 | 121126140 |
| #14 | *TBX15* | Missense | NM_152380 | C>T | A208T | chr1 | 119441735 |
| #15 | *BSN* | Missense | NM_003458 | G>A | R3672Q | chr3 | 49700606 |
| #15 | *CSMD2* | Missense | NM_052896 | C>T | V808I | chr1 | 34191103 |
| #15 | *DEPDC5* | Missense | NM_001136029 | G>A | V364M | chr22 | 32200156 |
| #15 | *DTNA* | Missense | NM_001198943 | T>C | C11R | chr18 | 32398199 |
| #15 | *FGF2* | Missense | NM_002006 | A>G | Q265R | chr4 | 123813478 |
| #15 | *LRRIQ1* | Missense | NM_001079910 | G>A | S20N | chr12 | 85432013 |
| #15 | *MRPL43* | Missense | NM_032112 | G>A | A3V | chr10 | 102747193 |
| #15 | *PKP4* | Missense | NM_001005476 | G>A | V489M | chr2 | 159490704 |
| #15 | *SLC15A1* | Missense | NM_005073 | C>T | A680T | chr13 | 99337067 |
| #15 | *TET2* | Nonsense | NM_001127208 | C>T | R544X | chr4 | 106156729 |
| #15 | *TLE1* | Missense | NM_005077 | C>T | R534H | chr9 | 84205948 |
| #15 | *TOP2B* | Splice site | NM_001068 | C>G | - | chr3 | 25677426 |
| #16 | *ACOX3* | Nonsense | NM_001101667 | C>T | W413X | chr4 | 8394121 |
| #16 | *ADRBK1* | Missense | NM_001619 | C>T | R591W | chr11 | 67052434 |
| #16 | *CSPG4* | Missense | NM_001897 | A>C | V1825G | chr15 | 75969386 |
| #16 | *GNPDA2* | Missense | NM_138335 | G>C | P149R | chr4 | 44713118 |
| #16 | *SHANK3* | Missense | NM_001080420 | C>T | P1266S | chr22 | 51160009 |
| #16 | *TSSK2* | Missense | NM_053006 | G>A | R59Q | chr22 | 19119088 |
| #17 | *HPR* | Nonsense | NM_020995 | C>T | Q186X | chr16 | 72110489 |
| #17 | *MAN2A2* | Missense | NM_006122 | G>T | R992L | chr15 | 91459467 |
| #17 | *MAP1S* | Missense | NM_018174 | C>T | R637C | chr19 | 17838102 |
| #17 | *MUC12* | Indel | NM_001164462 | */-ACT | 464_465del | chr7 | 100635235 |
| #17 | *PEAK1* | Missense  /splice site | NM_024776 | C>T | G1111E | chr15 | 77426092 |
| #17 | *RRM1* | Missense | NM_001033 | G>A | R648Q | chr11 | 4154830 |
| #17 | *RUNX1* | Nonsense | NM_001001890 | G>T | S114X | chr21 | 36252940 |
| #18 | *C12orf51* | Missense | NM_001109662 | C>A | C1776F | chr12 | 112673067 |
| #18 | *COL4A3* | Missense | NM_000091 | A>T | K1579N | chr2 | 228174016 |
| #18 | *EML3* | Missense | NM_153265 | G>C | N805K | chr11 | 62370303 |
| #18 | *OR51A7* | Missense | NM_001004749 | C>A | S74Y | chr11 | 4928820 |
| #18 | *POM121L2* | Missense | NM_033482 | G>A | P131S | chr6 | 27279559 |
| #18 | *PRCC* | Missense | NM_005973 | T>C | Y360H | chr1 | 156756961 |
| #18 | *PRDM9* | Indel | NM_020227 | */-AGA | 11_12del | chr5 | 23509175 |
| #18 | *RIC3* | Missense | NM_001135109 | C>T | R33Q | chr11 | 8190439 |
| #19 | *DYSF* | Missense | NM_001130976 | G>A | R2027Q | chr2 | 71909725 |
| #19 | *MMP11* | Indel | NM_005940 | */-C | P433fs | chr22 | 24124634 |
| #19 | *FAM120A* | Missense | NM_014612 | C>T | H254Y | chr9 | 96238576 |
| #19 | *FBXW4* | Missense | NM_022039 | C>T | G6R | chr10 | 103454382 |
| #19 | *KIAA1755* | Missense | NM_001029864 | G>T | T1157N | chr20 | 36841577 |
| #19 | *MDN1* | Nonsense | NM_014611 | G>A | Q2378X | chr6 | 90422953 |
| #19 | *OTOF* | Missense | NM_194322 | T>C | Y329C | chr2 | 26698297 |
| #19 | *PCDH11Y* | Missense | NM_032973 | G>A | R1262H | chrY | 5605745 |
| #20 | *ABCA13* | Missense | NM_152701 | T>C | V1663A | chr7 | 48314251 |
| #20 | *BBS9* | Missense | NM_001033604 | T>C | I305T | chr7 | 33313466 |
| #20 | *CSMD2* | Missense | NM_052896 | G>A | T27M | chr1 | 34554782 |
| #20 | *EXTL2* | Missense | NM_001033025 | C>T | V183I | chr1 | 101339944 |
| #20 | *FHOD3* | Missense | NM_025135 | T>C | F694L | chr18 | 34297866 |
| #20 | *OR5AU1* | Missense | NM_001004731 | G>C | D172E | chr14 | 21623669 |
| #20 | *PISD* | Missense | NM_014338 | C>T | E182K | chr22 | 32017371 |
| #20 | *SLK* | Missense | NM_014720 | C>T | R891C | chr10 | 105768001 |
| #21 | *DHX32* | Missense | NM_018180 | A>G | M377T | chr10 | 127541174 |
| #21 | *DOCK6* | Missense | NM_020812 | T>A | D1181V | chr19 | 11332535 |
| #21 | *DYSF* | Missense | NM_001130976 | G>A | R1663H | chr2 | 71891541 |
| #21 | *HHIPL2* | Splice site | NM_024746 | C>T | G526_splice | chr1 | 222711989 |
| #22 | *C12orf66* | Missense | NM_152440 | C>G | V214L | chr12 | 64588320 |
| #22 | *CACNA1E* | Missense | NM_001205294 | G>A | R1331H | chr1 | 181725151 |
| #22 | *CENPF* | Missense | NM_016343 | C>T | P2993L | chr1 | 214832208 |
| #22 | *FUCA2* | Nonsense | NM_032020 | G>A | R374X | chr6 | 143823103 |
| #22 | *TTC39A* | Missense | NM_001080494 | A>G | Y355H | chr1 | 51761836 |
| #22 | *UCHL5* | Missense | NM_001199261 | T>C | I35M | chr1 | 193020919 |
| #23 | *ASCC3* | Splice site | NM_006828 | A>G | - | chr6 | 101091905 |
| #23 | *CCKBR* | Missense | NM_176875 | G>A | V69I | chr11 | 6290952 |
| #23 | *DNAH3* | Missense | NM_017539 | T>C | S2817G | chr16 | 20981123 |
| #23 | *GRIN2A* | Missense | NM_001134407 | G>T | Q983K | chr16 | 9858454 |
| #23 | *IQUB* | Missense | NM_178827 | C>T | G312S | chr7 | 123142740 |
| #23 | *KDM1A* | Missense | NM_015013 | C>T | T835M | chr1 | 23409802 |
| #23 | *LTBP1* | Missense | NM_001166264 | G>A | G1011R | chr2 | 33585798 |
| #23 | *PCSK9* | Missense | NM_174936 | G>A | V296I | chr1 | 55521752 |
| #23 | *SERPINB13* | Missense | NM_012397 | G>A | V290M | chr18 | 61264289 |
| #23 | *TMC3* | Missense | NM_001080532 | A>G | I523T | chr15 | 81636337 |
| #24 | *CENPE* | Missense | NM_001813 | T>G | N429T | chr4 | 104098160 |
| #24 | *CLSTN2* | Missense | NM_022131 | G>T | Q362H | chr3 | 140178475 |
| #24 | *DSG4* | Missense | NM_001134453 | C>T | S113L | chr18 | 28968451 |
| #24 | *KIAA1522* | Missense | NM_001198972 | C>T | R336C | chr1 | 33235963 |
| #24 | *NR3C1* | Missense | NM_000176 | A>G | W600R | chr5 | 142678327 |
| #24 | *R3HDM1* | Missense | NM_015361 | T>G | L1004R | chr2 | 136481573 |
| #24 | *TGFBR3* | Missense | NM_001195683 | G>A | T707M | chr1 | 92177843 |
| #24 | *TPGS2* | Missense | NM_015476 | T>C | K100E | chr18 | 34385421 |
| #24 | *WNK2* | Missense | NM_006648 | C>T | T2122M | chr9 | 96070715 |

Chr, chromosome; Ref, reference

**Supplementary Table S2. Results of GO analysis with p value < 0.01**

| GO ID | GO term | Gene | Total gene number | Selected gene number | p-value |
| --- | --- | --- | --- | --- | --- |
| 5509 | calcium ion binding | *PCDHA10, RPH3A, DTNA, VWCE, PLCD1, PCDH11Y, RUNX1, DGKG, NPNT, TPPP, PCLO, GPR98, MMP11, OTOF, EHD1, TTN, LTBP1, CLSTN2, DLK2, FAT3,DSG4* | 668 | 21 | 1.E-05 |
| 5796 | Golgi lumen | *CSPG4,MUC2,MMP11,NGF,MUC12, BCAN* | 71 | 6 | 1.E-05 |
| 6941 | Striated muscle  contraction | *DTNA,MYH6,TTN* | 15 | 3 | 5.E-04 |
| 30512 | Negative regulation of TGF-beta receptor  signaling pathway | *TGFBR3,UCHL5,NR3C1,PPP1R15A, HTRA1* | 62 | 5 | 5.E-04 |
| 70579 | Methylcytosine dioxygenase activity | *TET2,TET3,* | 4 | 2 | 7.E-04 |
| 45746 | Negative regulation of Notch signaling  pathway | *HEY2,DLK1,DLK2* | 17 | 3 | 7.E-04 |
| 60716 | Labyrinthine layer blood vessel development | *AKT1,PLCD1,HEY2* | 18 | 3 | 9.E-04 |
| 45214 | Sarcomere organization | *FHOD3,MYH6,TTN* | 20 | 3 | 1.E-03 |
| 45202 | Synapse | *DTNA,ADRBK1,PCLO,MAP1S,BCAN,TG* | 111 | 6 | 1.E-03 |
| 48739 | Cardiac muscle fiber development | *MYH6,TTN* | 7 | 2 | 2.E-03 |
| 51382 | Kinetochore assembly | *CENPE,CENPF* | 8 | 2 | 3.E-03 |
| 30030 | Cell projection  organization | *AKT1,IQUB,DNAH9* | 26 | 3 | 3.E-03 |
| 22008 | Neurogenesis | *GRIN2A,WDR62,PCSK9* | 29 | 3 | 4.E-03 |
| 940 | Condensed chromosome outer kinetochore | *CENPE,CENPF* | 9 | 2 | 4.E-03 |
| 4435 | Phosphatidylinositol phospholipase C activity | *PLCD1,CASR,CCKBR* | 30 | 3 | 4.E-03 |
| 5524 | ATP binding | *AKT1,DHX32,MDN1,SRCAP,ABCA13,PGK1,RUNX1,DGKG,DNAH3, ADRBK1,MYO1B,NDUFA13,TSSK2, DDX27,ACTL6A,MYH1,MSH6,ABCC9,MYH6,WNK2,CENPE,EHD1,SPEF2, SLK,TTN,RRM1,DNAH9* | 1477 | 27 | 4.E-03 |
| 42640 | Anagen | *AKT1,DSG4* | 10 | 2 | 5.E-03 |
| 43623 | Cellular protein complex assembly | *OOEP,RIC3* | 10 | 2 | 5.E-03 |
| 30208 | Dermatan sulfate biosynthetic process | *CSPG4,BCAN* | 11 | 2 | 6.E-03 |
| 60317 | Cardiac epithelial to mesenchymal transition | *TGFBR3,HEY2* | 11 | 2 | 6.E-03 |
| 60644 | Mammary gland Epithelial cell differentiation | *AKT1,FGF2* | 12 | 2 | 7.E-03 |
| 16887 | ATPase activity | *MDN1,ABCA13,MSH6,MYH6,DNAH9* | 115 | 5 | 8.E-03 |
| 5858 | Axonemal dynein  complex | *DNAH3,DNAH9* | 13 | 2 | 8.E-03 |
| 60216 | Definitive hemopoiesis | *TGFBR3,RUNX1* | 13 | 2 | 8.E-03 |
| 16079 | Synaptic vesicle  exocytosis | *PCLO,OTOF* | 14 | 2 | 1.E-02 |
| 31011 | Ino80 complex | *UCHL5,ACTL6A* | 14 | 2 | 1.E-02 |
| 5604 | Basement membrane | *COL7A1,COL4A3,RUNX1,NPNT* | 78 | 4 | 1.E-02 |
